# Focusing on microvascular function in heart failure with preserved ejection fraction

**DOI:** 10.1007/s10741-024-10479-7

**Published:** 2025-01-13

**Authors:** Ornela Velollari, Karl-Philipp Rommel, Karl-Patrik Kresoja, Philipp Lurz, Tommaso Gori

**Affiliations:** 1 Department of Cardiology, Cardiology I, University Medical Center Mainz, Langenbeckstrasse 1, 55131 Mainz, Germany; 2 German Centre for Cardiovascular Research (DZHK), Standort Rhein-Main, Frankfurt, Germany

**Keywords:** Coronary microvascular dysfunction, Heart failure, Ischemia, Therapy, Coronary sinus reducer

## Abstract

Heart failure is a prevalent global health issue. Heart failure with preserved ejection fraction (HFpEF), which already represents half of all heart cases worldwide, is projected to further increase, driven by aging populations and rising cardiovascular risk factors. Effective therapies for HFpEF remain limited, particularly due to its pathophysiological heterogeneity and incomplete understanding of underlying pathomechanisms and implications. Coronary microvascular dysfunction (CMD), characterized by structural and functional changes in the coronary microcirculation, is increasingly recognized as a significant factor in HFpEF even though the exact nature of their causal relationship is still unclear. This review explores prevalence, prognostic implications, and potential therapeutic targets for CMD in HFpEF. CMD’s role in HFpEF might involve impaired coronary blood flow regulation, leading to myocardial ischemia, impaired relaxation, and/or adverse remodeling. Vice versa, increased wall stress in patients with HFpEF might elevate coronary resistances, further worsening microvascular perfusion. Finally, abnormalities in substrate metabolism might cause both CMD and HFpEF. Current treatments, including pharmacotherapy and device-based therapies, show limited success, highlighting the need for more targeted approaches. New possible therapies, such as the coronary sinus reducer device, may show promise in improving myocardial perfusion and function. However, further large-scale studies are required to elucidate the mechanistic links between CMD and HFpEF and to develop specialized treatments for distinct heart failure phenotypes.

## Introduction

Heart failure is a global pandemic, affecting more than 60 million people worldwide, with an age-dependent increasing incidence in Europe of 5/1000 patients per year [1–6]. Heart failure with preserved ejection fraction (HFpEF) makes up approximately half of all heart failure cases and is projected to be the dominant phenotype in the aging population due to the increasing prevalence of cardiovascular risk factors such as obesity, diabetes mellitus, hypertension, and atrial fibrillation [7, 8]. The pathophysiological heterogeneity of HFpEF hinders an adequate therapeutic solution for all HFpEF patients with the current therapeutic possibilities being limited to sodium-glucose transporter 2 inhibitors (SGLT-2 inhibitors) [9].

For a long time, left ventricular (LV) diastolic dysfunction was regarded as the hallmark of HFpEF, among other factors such as fibrosis, inflammation, ventricular-arterial stiffening, and atrial dysfunction. However, contractile dysfunction as manifested by global longitudinal strain has been recently suggested as a significant factor associated with HFpEF, also in subjects with preserved ejection fraction [10]. Notably, the high prevalence of subendocardial ischemia suggests a state of energy deficiency which could explain the observed impairments in both active (ATP-dependent) relaxation and systolic function.

Coronary artery disease (CAD) is highly prevalent in HFpEF [11], and the presence of CAD predisposes to the development of HFpEF [12]. The research focus until now has been mainly on the macrocirculation and epicardial branches, but recently there has been a shift towards the microcirculation. Endothelial dysfunction, associated with impaired peripheral perfusion, has been proposed as a possible mechanistic link between coronary microvascular dysfunction (CMD) and the pathogenesis of HFpEF [13]. This review addresses the prevalence of CMD in patients with HFpEF, its prognostic implications, and the potential target treatment.

## Pathophysiology of coronary microvascular dysfunction in HFpEF

**Fig. 1:**
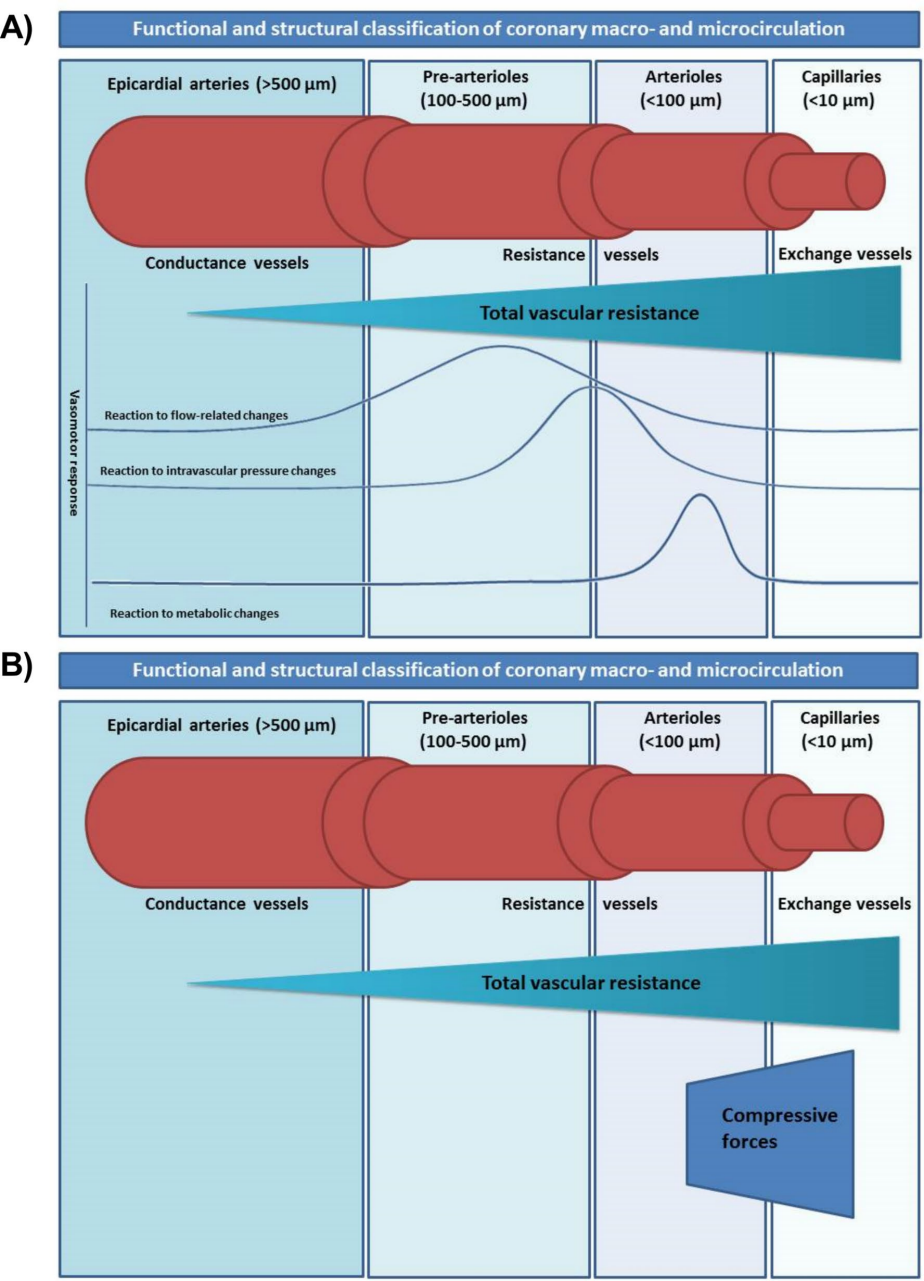
**A, B** Functional and structural classification of coronary macro- and microcirculation in healthy cohorts and functional changes in heart failure. **A** Pre-arterioles (< 500 µm in diameter) are epicardial, extra-myocardial vessels that react to changes in shear stress, and intravascular pressure to maintain the coronary downstream flow. Arterioles (< 100 µm in diameter) are intra-myocardial vessels that regulate coronary microcirculation. Depending on the diameter, a different response to pressure or flow stimuli reigns. Vessels with a diameter of 100–200 µm translate flow-related stimuli into a vasomotor response (vasodilation or vasoconstriction), and vessels with a diameter between 40 and 100 µm react to changes in pressure to modulate coronary flow, whereas vessels < 40 µm in diameter react to metabolic changes (represented by the heightened curve of reaction to metabolic changes) of the myocardium with subsequent vasodilation or vasoconstriction. **B** In the presence of heart failure, compressive extra-/intraymyocardial forces are heightened due to structural changes (fibrosis, concentric remodeling) and/or elevated LV-filling pressure, which exert an external pressure on the subendocardial capillaries and arterioles and subsequently lead to compression of coronary microvessels, increase of vascular resistance, decrease of coronary vascular flow, imbalance between myocardial oxygen supply and demand, and therefore ischemia

The coronary vasculature is made of capacitance and resistance vessels. Epicardial arteries and the venous system are capacitance vessels, offering little vascular resistance (~ 10%). Pre-arterioles, arterioles are resistance vessels, being responsible for the remaining 75–80% of the coronary vascular resistance (Fig. 1A). Capillaries and venules contain around 90% of the total myocardial blood volume, and their role in determining total vascular resistance has long been disregarded. Pre-arterioles and arterioles regulate coronary microcirculation by translating flow- or pressure-related stimuli (changes in intravascular pressure, coronary flow, or metabolic changes) into a vasomotor response to maintain the coronary downstream flow [14, 15]. The capillary bed, which currently cannot be targeted pharmacologically, also contributes to total vascular resistance, especially under conditions of increased wall stress.

Microvascular dysfunction comprises structural and functional changes of the coronary microcirculation due to an array of cardiovascular risk factors and cardiac pathologies such as aortic stenosis, amyloidosis, heart failure, epicardial obstruction, and cardiomyopathies. CMD can be defined as a limited coronary vasodilation and/or abnormally high vascular resistance (or even paradoxical vasoconstriction) in response to physiological or pharmacological stimuli. To date, CMD (both endothelium-dependent and -independent) encompasses several phenotypes, including microvascular angina with reduced coronary flow reserve (CFR) and/or elevated microvascular resistance (MR), microvascular spasm, and epicardial dysfunction (epicardial spasm). Similar to HFpEF, CMD is an overarching term to indicate a number of endotypes, which poses challenges for effective therapy management.

Fundamentally, coronary blood flow is driven by the pressure difference between the aortic sinus and the coronary sinus, which equals the right atrial pressure. The alterations in the myocardial microcirculation affect myocardial perfusion and can lead to ischemia, irrespective of epicardial obstructions. Moreover, disturbances in coronary autoregulation impair blood flow responses to increased oxygen demand. The main pathophysiological processes that alter coronary blood flow and/or resistance leading to CMD may include extravascular compression (restricted diastolic perfusion time, elevated cardiac metabolism, and intramyocardial compressive forces), vascular dysfunction (of endothelial and vascular smooth cells), and vaso-structural changes (vascular-wall infiltration, remodeling, obstruction, rarefaction, and perivascular fibrosis). Invasive hemodynamic examination of patients with HFpEF and HFrEF showed similar prevalence rates of CMD in both subgroups with different patterns in changes of (absolute) microvascular resistance and myocardial flow, possibly supporting the concept that HF, independently of its cause, may lead to (different forms of) CMD [16]. Postmortem analysis of myocardial tissue of HFpEF patients has revealed rarefaction of the coronary microvasculature correlating to myocardial fibrosis [17]. Inflammation, an important pathophysiological process, leading to endothelial dysfunction, has also been proposed to mediate the development of CMD in HFpEF. Endothelial dysfunction and the consequent imbalance between reduced bioavailability of nitric oxide (NO) and increased production of vasoconstrictors decrease perfusion and increase myocardial tension. Also, decreased NO-dependent activation of protein kinase G (PKG) leads to decreased phosphorylation of titin, an important structural component for diastolic myocyte recoil and flexibility during diastole [18–20]. HFpEF patients show higher collagen type I and cross-linking associated with diastolic dysfunction, driven by inflammation-induced fibroblast activation and collagen deposition [21, 22].

**Fig. 2:**
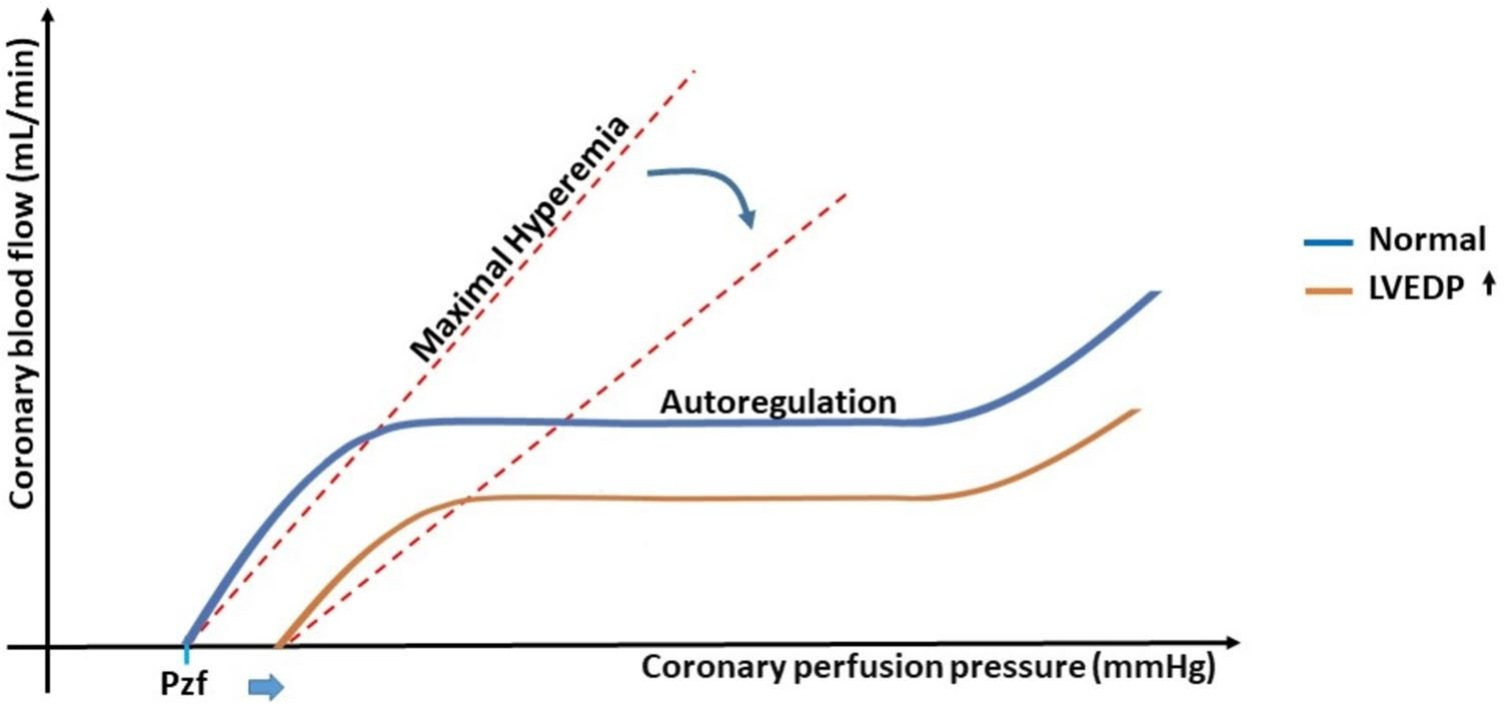
Changes in coronary flow and pressure in the presence of elevated left ventricular end-diastolic pressure (LVEDP). Illustrative depiction of coronary pressure-flow relationship demonstrating coronary autoregulation under normal conditions and at elevated LVEDP. In case of heart failure with elevated LVEDP, intercept of pressure zero flow (Pzf) is shifted to the right, with a reduction of coronary flow at max hyperemia

Cardiac damage occurs predominantly in the subendocardial layer due to the summation of compressive forces, increased left ventricular end-diastolic pressure, and changes in epicardial conductance, which collectively reduce coronary flow and shift the oxygen supply/demand relationship (Fig. 1B). The subendocardial blood flow at maximal coronary vasodilation is determined by the duration of the diastole and the differences in driving pressures. Under physiological conditions, the driving pressure of the coronary blood flow is the difference in aortic diastolic pressure to zero-flow pressure, which is believed to be close to central venous pressure. However, when left ventricular (LV) diastolic pressure is elevated, it becomes the driving antagonistic pressure to the aortic diastolic pressure, increasing zero-flow pressure above the venous pressure level and reducing coronary perfusion (Fig. 2). In HFpEF, concentric remodeling, resulting in increased wall thickness induced by chronic pressure overload, can increase oxygen demand and lead to extravascular compression of the coronary microvessels, thus reducing coronary flow reserve and eventually leading to an imbalance between myocardial oxygen supply and demand [23]. There is a correlation between increasing LV filling pressures and decreasing coronary flow reserve [24]. Another pathophysiological aspect in HFpEF that can specifically reduce hyperemic diastolic coronary flow is the delay in LV relaxation and therefore in coronary filling.

**Fig. 3:**
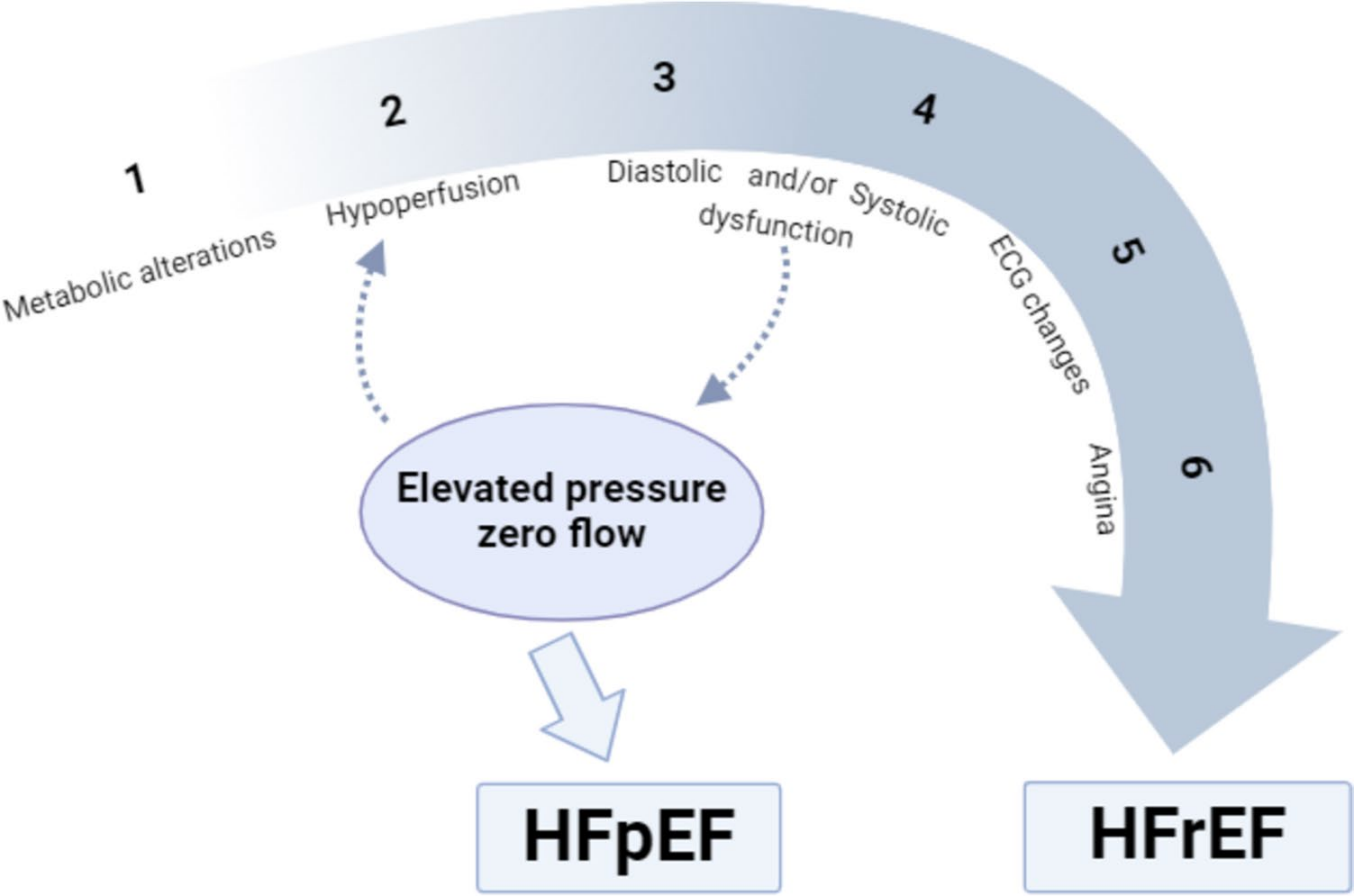
Ischemia pattern in the case of heart failure following a variety of temporal sequences consisting of hemodynamic, electrocardiographic, and symptomatic expressions of ischemia

Conversely, these processes might perpetuate each other as relative myocardial ischemia hampers active (ATP-dependent) relaxation, with the latter being one of the earliest detectable signs in the ischemia cascade (Fig. 3). To summarize, LV diastolic dysfunction could both contribute to and be a consequence of subendocardial ischemia [23]. As such, ascribing causality is problematic. While it is likely that HFpEF deteriorates CMD and vice versa, it remains elusive if CMD patients have an elevated propensity to develop HFpEF over time. Ultimately, hypoperfusion may lead to impaired contractility and HFrEF.

### Diagnosis

Given the overlap in the clinical presentation of HFpEF and CMD, their common mechanisms, and the implications for therapy, it is important that patients with a diagnosis of HFpEF undergo screening for CMD (and vice versa). Patients diagnosed with HFpEF can initially be tested with non-invasive modalities such as cardiac positron emission tomography (PET), cardiac magnetic resonance imaging (CMR), which allow for assessing the myocardial blood flow (MBF) in all coronary territories at rest and hyperemia and the global myocardial perfusion reserve/-index (MPR/MPRI) [25, 26] and therefore provide information on the vasodilatory capacity of the microcirculation. Transthoracic Doppler echocardiography (TTDE) can also be used in selected centers to measure the coronary flow velocity ratio (CFVR), a surrogate of CFR. Usually, a cut-off value of less than 2.0 for CFVR, MBF, and MPR/MPRI hints towards an impaired vasodilatory capacity, confirming CMD in a patient who has no obstructive CAD [27]. We believe that all patients with HFpEF should undergo this type of investigation. Alternatively, in patients undergoing coronary angiography, invasive methods such as intracoronary Doppler flow-pressure wire or temperature pressure wire (with bolus-based/continuous thermodilution method) can examine not only coronary flow at rest and hyperemia, but also provide a measure of microvascular resistance. A reduced CFR (< 2.5) in the absence of epicardial CAD (FFR ≥ 0.8), reduced microvascular resistance reserve (MRR) (< 2.7) and/or an elevated IMR (≥ 25 U), and hyperemic microvascular resistance (HMR) (> 2.5 mmHg/cm/s) can be indicative of microvascular dysfunction [27–29]. Recent diagnostic methods such as the continuous thermodilution method can assess absolute flow (*Q*) and resistance (*R*µ) without the use of hyperemic agents, with greater diagnostic accuracy and superior repeatability [30, 31]. Invasive wireless methods such as coronary angiography-based IMR (ca-IMR) can also apprehensively evaluate microvascular resistance, although randomized trials are necessary to confirm the validity of ca-IMR and establish cut-off values [32, 33].

The current 2024 ESC Guidelines on assessing CMD in patients with HFpEF give a class IIa recommendation for the investigation of CMD in HFpEF (via positron-emission tomography or magnetic resonance). This recommendation will hopefully raise the interest of clinicians in this field and will provide a better understanding of CMD’s role in HFpEF in the future.

Biomarker studies in INOCA and CMD have identified a range of circulating biomarkers linked to inflammation (e.g., hs-CRP, Interleukin-1, Interleukin-6), oxidative stress (e.g., glutathione, superoxide free radicals, cystine), coagulation (e.g., von Willebrand factor, D-dimer, fibrinogen), and endothelial dysfunction (e.g., endothelin-1, lipoprotein (a), renalase) [34]. Traditional biomarkers such as Troponin I (TnI), NT-pro BNP and B-type natriuretic peptide (BNP), and signaling myocardial damage have been shown to be associated with risk for major adverse cardiovascular events in patients with impaired CFR [10, 35]. NT-pro BNP, compared to BNP, has greater stability with a longer half-life concentration and is not influenced using neprilysin inhibitors [36, 37]. The upcoming WISE pre-HFpEF (NCT03876223) study is set out to investigate how CMD-related ischemia contributes to myocellular damage, impaired ventricular relaxation, and diastolic dysfunction progression, by directly assessing TnI from coronary sinus/great cardiac vein.

Similarly, HFpEF patients exhibit elevated levels of CMD-related biomarkers, with notable sex-dependent differences: women show higher levels of biomarkers associated with fibrosis-signaling pathways, while men exhibit more inflammation-related biomarkers [38]. Elevated systemic oxidative stress markers, such as cystine (but not glutathione), have been associated with diastolic dysfunction in patients with INOCA, highlighting the physiological relationship between CMD and HFpEF [39]. Pregnancy-associated plasma protein A (PAPP-A), a novel biomarker linked to reduced CFR in HFpEF and predictive of cardiovascular events in acute coronary syndrome (ACS), underscores the role of subclinical atherosclerosis in the pathophysiology of CMD and HFpEF [40]. However, the diagnostic role of biomarkers for CMD is less established and requires further clinical validation.

On the other side, we also believe that all patients with confirmed CMD should undergo screening for HFpEF, including functional assessment, transthoracic echocardiography, and biomarker assessment.

### Prevalence and clinical implications of coronary microvascular dysfunction in HFpEF

Patients with HFpEF and without epicardial obstruction display a reduced myocardial flow rate (MFR) in cardiac PET compared to normal controls or hypertensive patients [41]. This raises the question of the prevalence and role of CMD in HFpEF. Since CMD is not systematically investigated in patients with HFpEF, we strongly believe that the prevalence of this condition is extremely underestimated. In studies that assessed CMD invasively, HFpEF patients with no epicardial obstruction had a prevalence of CMD (endothelial-dependent and -independent) as high as 81% [10, 24, 42–44] and an increased risk for cardiac death or heart failure hospitalization [43, 45, 46]. In meta-analyses, the pooled prevalence of CMD in patients with HFpEF ranged between 55.5 and 71%, and among the five studies that provided prognostic data, in four of them, the presence of CMD was associated with poor clinical outcomes, such as cardiac death and heart failure hospitalization [47, 48]. The presence of CMD in HFpEF patients was further associated with echocardiographic evidence of worse diastolic dysfunction, higher prevalence of atrial fibrillation, and increased risk of death and HF hospitalization [49]. Similarly, the presence of CMD in HFpEF is associated with reduced left atrial, left ventricular, and right ventricular strain [10], underlining the pathophysiological relationship between subendocardial ischemia and CMD. Patients with CMD (CFR < 2) and E/e′ > 15 had a significantly higher incidence of hospitalization due to HFpEF compared to patients with CMD and normal ventricular filling pressure, hinting towards an association between myocardial ischemia due to CMD and diastolic dysfunction, the main underlying pathology of HFpEF [50].

### Therapy

HFpEF patients usually receive treatments for cardiovascular risk factors and/or amelioration of accompanying symptoms of heart failure. Among others, these mostly include pharmacotherapy comprising SGLT-2 inhibitors and/or mineral corticoid receptor antagonists, which improve quality of life and reduce hospitalization [9, 51]. Nonetheless, patients with HFpEF have a high rate of rehospitalization after their initial diagnosis [52]. Similarly, therapeutic options in CMD are limited, and studies, particularly those investigating a given intervention in specific endotypes of CMD, are missing. Common cardiovascular risk factors, such as diabetes mellitus, hypertension, obesity, and dyslipidemia, along with lifestyle factors like smoking, weight management, and physical activity, are known to significantly affect endothelial function [53–57]. Evidence indicates that weight loss and regular physical exercise can reduce angina symptoms, enhance coronary blood flow, and improve exercise capacity [58, 59]. While smoking cessation is believed to positively impact endothelial function, the current evidence supporting this claim is limited. Pharmacotherapy has had little success in considerable symptom amelioration and major adverse cardiovascular event (MACE) reduction. Calcium-channel and beta-blockers have been shown to modify endothelial function and improve anginal symptoms [60], although randomized controlled large-scale trials are necessary to determine the therapeutic efficacy. Pharmaceuticals targeting elevated endothelin-1 (ET-1) levels in CMD patients, such as ET-1 receptor antagonists (darusentan and atrasentan), demonstrated improvements in coronary flow in small-scale randomized trials [61, 62]. However, these results were not replicated in subsequent phase III trials. Similarly, macitentan and newer agents like zibotentan failed to show benefits in reducing angina burden or increasing mean exercise duration and were associated with a higher incidence of adverse events, including headaches, peripheral edema, nasopharyngitis, and breathlessness [63, 64]. Nitric oxide pathway modulators (sildenafil, cilostazol) have shown promise in small randomized trials, improving angina burden and CFR in patients with microvascular and epicardial dysfunction [65, 66]. Nonetheless, larger and longer-term studies are required to validate these findings. In the VICTORIA trial [67] Vericiguat showed similar treatment benefit on the composite endpoint (cardiovascular death, heart failure hospitalization, stroke and myocardial infarction) in both the coronary artery disease (CAD) and no CAD group for patients with heart failure with reduced ejection fraction (HFrEF). Following up on this observation, the V-COM trial (NCT06239974) is an upcoming randomized controlled trial which plans to determine the effectiveness of vericiguat in improving angina symptoms assessed by Seattle Angina Questionnaire (SAQ)−7, stress myocardial blood flow, and myocardial perfusion reserve as measured by cardiac magnetic resonance imaging. The COL-MICRO-HF (NCT06217120) is an upcoming study, aiming to evaluate the impact of colchicine on CFR in patients with HF and ejection fraction ≥ 40% compared to placebo. Device-based therapy such as coronary sinus reducer has been associated with an improvement in angina class and frequency, a reduction of microvascular resistance, and myocardial perfusion changes [68–71]. An effect specifically in HFpEF patients has not been demonstrated yet.

### Cell-mediated therapy

Intracoronary infusion of autologous CD34 + -marked endothelial progenitor cells improved coronary flow reserve, angina frequency, and life quality in patients with refractory angina and endothelium-independent CMD at 6 months after treatment. CD34 + -marked cells induce capillary growth, promote vascular repair in ischemic areas, and enhance angiogenesis, without serious cell-related adverse events [72]. A similar approach in a randomized controlled trial in patients with nonischemic dilated cardiomyopathy led to improvements in myocardial perfusion and left ventricular ejection fraction at 6 months after therapy [73]. Transendocardial injection of CD34 + -marked cells in patients with HFpEF led to improvements in diastolic parameters and exercise capacity (improvement in E/e′, NT‐proBNP, exercise capacity, and maximal tricuspid regurgitation velocity) without significant changes of the global systolic function, but rather local systolic function at the site of injections [74]. CMD and HFpEF patients may have limited benefits from pharmacotherapy partially due to advanced structural and microvascular changes and partially due to heterogeneous phenotypes grouped together. The studies mentioned may suggest that CD34 + -cell therapy could be a potential treatment targeting both conditions. Results of such studies focusing on CMD in HFpEF patients or vice versa are yet to be published.

### Device-based therapy

A coronary sinus reducer is a balloon-expandable, hourglass-shaped, stainless steel mesh, which narrows the coronary sinus and increases coronary venous pressure, which might lead to redistribution of collateral blood flow from nonischemic into ischemic territories of the myocardium, reduce myocardial ischemic damage, and alleviate angina symptoms. The 2024 ESC Guidelines on chronic coronary syndrome (recommendation class IIb) rate CS-reducer as an alternative therapy for patients with refractory angina, who have exhausted all options for medical therapy and are not candidates for further revascularization by percutaneous coronary intervention (PCI) or coronary artery bypass graft (CABG) surgery [75].

**Fig. 4:**
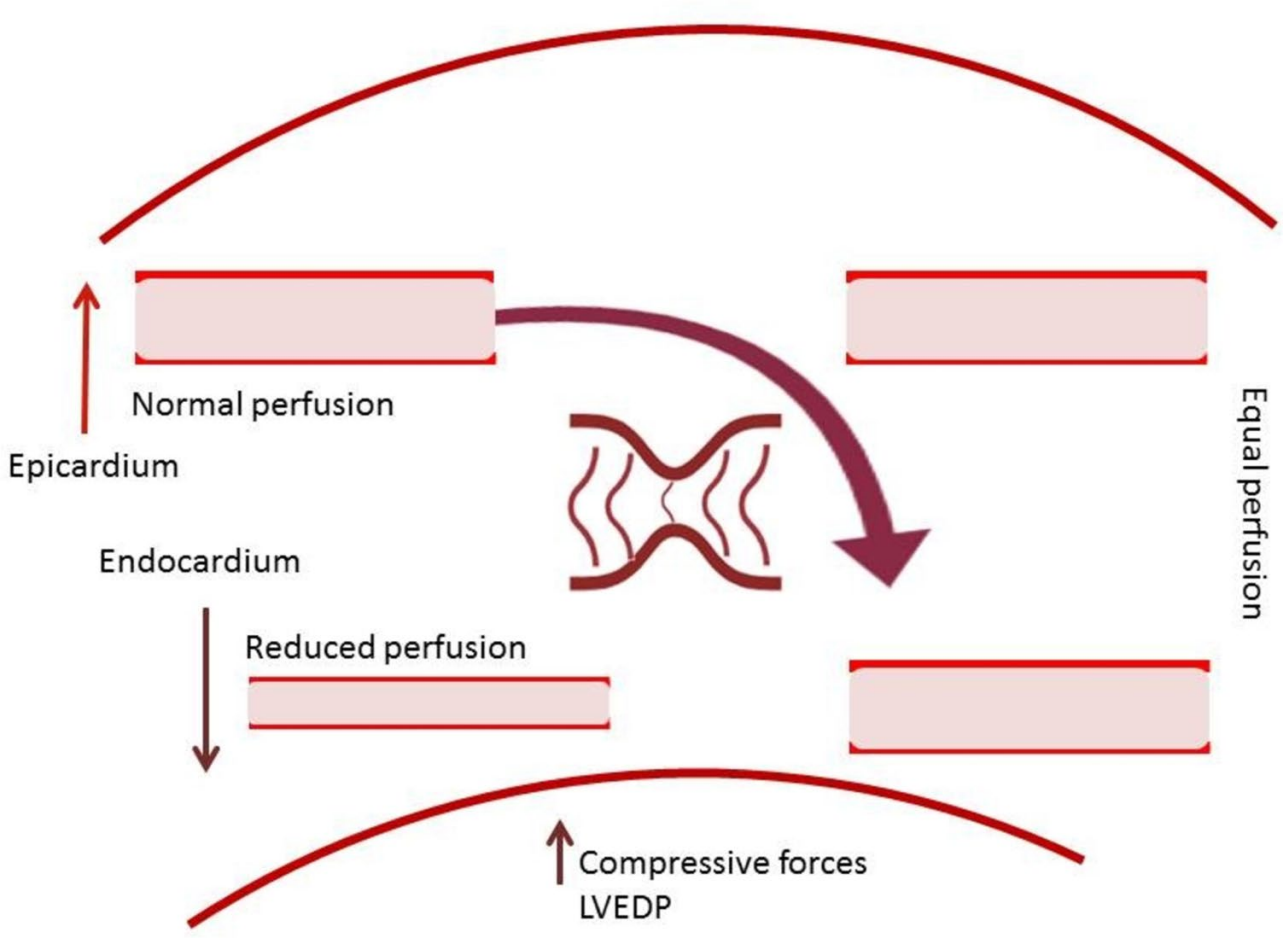
Presumed mechanistic effect of the coronary sinus reducer on myocardial flow and epi-/endocardial layers. In the case of subendocardial ischemia, different tissue pressures across the left ventricular wall restrict subendocardial perfusion, while subepicardial layers maintain preserved perfusion, leading to transmural (vertical) microvascular steal [80]. Coronary sinus occlusion increases coronary venous pressure, presumably leading to a dilation of the subendocardial arterioles and a reduction of vascular resistance in the subendocardium. Consequently, blood flow and contractility in the ischemic subendocardial layers are possibly improved and left ventricular end-diastolic pressure (LVEDP) reduced [81, 82]. The coronary sinus reducer is presumed to correct the microvascular steal by equalizing the blood flow across the layers of the left ventricular wall, from subepicardial to subendocardial, shifting flow from nonischemic to ischemic myocardial regions to improve ischemia

The principal effect of the CSR is thought to be mediated by an improvement in subendocardial perfusion caused by either increased capillary transit time or redistribution of flow from non-ischemic to ischemic regions, a hypothesis particularly relevant in the presence of stenosis and possibly in the setting of increased wall tension [76]. A study using Rubidium-82 after CSR implantation in patients with refractory angina confirmed a redistribution of myocardial blood flow from better to less-perfused areas as a mechanistic effect of CSR and an improvement in myocardial perfusion reserve index and angina levels in less-perfused myocardial areas mediated by changes in stress myocardial blood flow [77]. An observational stress CMR study after CSR implantation [78] could demonstrate an amelioration of transmural myocardial perfusion with a focus on subendocardium (Fig. 4), improvement of left ventricular contractility, circumferential and longitudinal strain, and reduction of ischemic burden, without changes in diastolic function, focal or interstitial fibrosis, and myocardial stiffness. Similarly, the ORBITA-COSMIC study demonstrated a redistribution of perfusion from subepicardial to subendocardial myocardium in ischemic segments [79].

Although data from the MACCUS trial [70] and later INROAD study [71] have shown promising results in patients with microvascular angina, a therapy with CS-reducer for CMD still needs more solid evidence. Ongoing studies such as COSIMA (NCT04606459) and COSIRA-II (NCT05102019) aim to confirm the efficacy of a device-based therapy in a large, randomized trial setting.

Evidence in the setting of HF is sparser. In a murine model, increased CS pressure led to interstitial myocardial edema and long-term epicardial fibrotic expansion [83], possibly due to reduced coronary blood flow, elevated coronary vascular resistance, and elevated LVEDP. However, this modification has not been observed in humans [78], and there is evidence that CSR implantation might lead to an overall improved myocardial performance during exercise, as evidenced by improvement in maximal oxygen consumption (VO2max) and oxygen consumption at the anaerobic threshold VO2_at_ during cardiopulmonary exercise testing and an improvement in left ventricular ejection fraction, specifically in the subgroup with reduced ejection fraction [84, 85]. In a small group (*N* = 24) of HF patients (96% with diastolic dysfunction grades I–III) [86], occlusion of coronary sinus by implanting a coronary sinus reducer led to a significant improvement of mean diastolic function class without adversely affecting diastolic function. Finally, experimental data have suggested that coronary flow modulation by temporary CS occlusion might exert pleiotropic effects (i.e., increase in micro-RNAs), which promote myocardial/endothelial cell proliferation and differentiation (cardiac morphogenesis) [87].

In sum, while promising for the treatment of CMD, data regarding the implication of CSR therapy on diastolic function are insufficient to draw any conclusion. Further large-scale studies are needed focusing on left ventricular systolic and diastolic function after CSR implantation.

## Conclusion

HFpEF and CMD are complex and heterogeneous syndromes with high overlap and limited therapeutic options. Their causal relationship remains incompletely understood. While the presence of HFpEF very likely aggravates CMD, cumulating evidence supports the notion of HFpEF being a syndrome of myocardial energy deprivation, and thus, CMD-related myocardial ischemia could well be a major contributor to HFpEF development. A device-based therapy approach for CMD with implantation of a sinus reducer can improve subendocardial perfusion, while hinting at improvement of diastolic function. As such, this therapy might be well positioned to alleviate hemodynamic and functional alterations in patients with HFpEF. Future studies should focus on the elucidation of pathophysiological links between CMD and HFpEF phenotypes in order to clarify whether CMD might be a specific therapeutic target in patients with HFpEF. In addition, investigations into the propensity of specific CMD phenotypes to develop HFpEF over time are needed in order to clarify whether CMD could also be a target for a preventive treatment approach.

## Data Availability

No datasets were generated or analyzed during the current study.

## Abbreviations

ACS: Acute coronary syndrome
AT: Anaerobic threshold
BNP: B-Type natriuretic peptide
CABG: Coronary artery bypass graft
CAD: Coronary artery disease
Ca-IMR: Coronary angiography-based IMR
CFR: Coronary flow reserve
CMD: Coronary microvascular dysfunction
CMR: Cardiac magnetic resonance imaging
CPET: Cardiopulmonary exercise testing
CRP: C-Reactive protein
CS: Coronary sinus
CSR: Coronary sinus reducer
CTCA: Computed tomography coronary angiography
ET-1: Endothelin-1
HF: Heart failure
HFpEF: Heart failure with preserved ejection fraction
HFrEF: Heart failure with reduced ejection fraction
HMR: Hyperemic microvascular resistance
IMR: Index of microvascular resistance
INOCA: Ischemia with non-obstructive coronary artery
LV: Left ventricle
LVEDP: Left ventricular end-diastolic pressure
MACE: Major adverse cardiovascular event
MBF: Myocardial blood flow
MFR: Myocardial flow rate
MPR: Myocardial perfusion reserve
MPRI: Myocardial perfusion reserve index
MRR: Microvascular resistance reserve
MVD: Microvascular dysfunction
NO: Nitric oxide
NT-pro BNP: N-Terminal pro-B-type natriuretic peptide
PAPP-A: Pregnancy-associated plasma protein A
PCI: Percutaneous coronary intervention
PET: Positron emission tomography
PKG: Protein kinase G
*Q*: Absolute flow
*R*µ: Absolute resistance
SAQ: Seattle Angina Questionnaire
SGLT-2: Sodium-glucose transporter 2
Troponin I: TnI
TTDE: Transthoracic Doppler echocardiography
VO2: Maximal oxygen consumption
